# Nitroxide Radical-Containing Redox Nanoparticles Protect Neuroblastoma SH-SY5Y Cells against 6-Hydroxydopamine Toxicity

**DOI:** 10.1155/2020/9260748

**Published:** 2020-04-24

**Authors:** Monika Pichla, Łukasz Pulaski, Katarzyna Dominika Kania, Ireneusz Stefaniuk, Bogumił Cieniek, Natalia Pieńkowska, Grzegorz Bartosz, Izabela Sadowska-Bartosz

**Affiliations:** ^1^Department of Analytical Biochemistry, Institute of Food Technology and Nutrition, College of Natural Sciences, Rzeszow University, Zelwerowicza Street 4, 35-601 Rzeszow, Poland; ^2^Department of Molecular Biophysics, Faculty of Biology and Environmental Protection, University of Lodz, Pomorska Street 141/143, 90-236 Lodz, Poland; ^3^Laboratory of Transcriptional Regulation, Institute of Medical Biology, Polish Academy of Sciences, Lodowa Street 106, 93-232 Lodz, Poland; ^4^Teaching and Research Center of Microelectronics and Nanotechnology, College of Natural Sciences, University of Rzeszow, Pigonia 1, 35-959 Rzeszow, Poland; ^5^Department of Experimental Physics, Teaching and Research Center of Microelectronics and Nanotechnology, College of Natural Sciences, University of Rzeszow, Pigonia 1, 35-959 Rzeszow, Poland

## Abstract

Parkinson's disease (PD) patients can benefit from antioxidant supplementation, and new efficient antioxidants are needed. The aim of this study was to evaluate the protective effect of selected nitroxide-containing redox nanoparticles (NRNPs) in a cellular model of PD. Antioxidant properties of NRNPs were studied in cell-free systems by protection of dihydrorhodamine 123 against oxidation by 3-morpholino-sydnonimine and protection of fluorescein against bleaching by 2,2-azobis(2-amidinopropane) hydrochloride and sodium hypochlorite. Model blood-brain barrier penetration was studied using hCMEC/D3 cells. Human neuroblastoma SH-SY5Y cells, exposed to 6-hydroxydopamine (6-OHDA), were used as an *in vitro* model of PD. Cells were preexposed to NRNPs or free nitroxides (TEMPO or 4-amino-TEMPO) for 2 h and treated with 6-OHDA for 1 h and 24 h. The reactive oxygen species (ROS) level was estimated with dihydroethidine 123 and Fluorimetric Mitochondrial Superoxide Activity Assay Kit. Glutathione level (GSH) was measured with *ortho*-phtalaldehyde, ATP by luminometry, changes in mitochondrial membrane potential with JC-1, and mitochondrial mass with 10-Nonyl-Acridine Orange. NRNP1, TEMPO, and 4-amino-TEMPO (25-150 *μ*M) protected SH-SY5Y cells from 6-OHDA-induced viability loss; the protection was much higher for NRNP1 than for free nitroxides. NRNP1 were better antioxidants *in vitro* and permeated better the model BBB than free nitroxides. Exposure to 6-OHDA decreased the GSH level after 1 h and increased it considerably after 24 h (apparently a compensatory overresponse); NRNPs and free nitroxides prevented this increase. NRNP1 and free nitroxides prevented the decrease in ATP level after 1 h and increased it after 24 h. 6-OHDA increased the intracellular ROS level and mitochondrial superoxide level. Studied antioxidants mostly decreased ROS and superoxide levels. 6-OHDA decreased the mitochondrial potential and mitochondrial mass; both effects were prevented by NRNP1 and nitroxides. These results suggest that the mitochondria are the main site of 6-OHDA-induced cellular damage and demonstrate a protective effect of NRNP1 in a cellular model of PD.

## 1. Introduction

Age-related diseases such as Parkinson's disease (PD) constitute a significant socioeconomic burden for modern populations. As human mean lifespan increases, increasing occurrence of PD has features of a pandemic. From 1990 to 2015, the number of people with this disease doubled worldwide to at least over 6 million. Driven mostly by increasing longevity, this number is foreseen to double again to over 12 million by 2040 [1]. Parkinson's disease is slowly progressive, and disease-modifying drugs are still not available. Parkinson's disease is a long-term degenerative disorder generally proceeding with motor and nonmotor symptoms [2, 3], which usually worsen with advancing age, leading to care dependency. Disease manifestation is indicated by the presence of Lewy bodies (abnormal protein aggregates predominantly containing *α*-synuclein, ubiquitin, Parkinson juvenile disease protein 2 (Parkin), PTEN-induced kinase-1 (PINK1), and other less abundant proteins) and death of dopaminergic neurons in the *substantia nigra* projecting to the striatum as well as microgliosis [4, 5]. Environmental as well as genetic factors (or unknown factors in idiopathic or sporadic cases) have been implicated in the mechanism underlying the pathogenesis of PD [6]. Nearly 10% of PD cases may be caused by mutations in over 12 different genes implicated in the regulation of proteasomal degradation pathways (Parkin, ubiquitin carboxy-terminal hydrolase L1), mitochondrial homeostasis (PINK1, mitochondrial serine protease Omi/Htra, integral mitochondrial protein DJ-1, and leucine-rich repeat kinase 2), lysosome function (ATPase cation transporting 13A2 (ATPase cation transporting 13A2)), antioxidant response pathways (oncogene DJ-1 also known as a Parkinson disease protein 7) and mitophagy (PINK1 and Parkin) [7, 8]. Nevertheless, currently, the molecular mechanisms underlying all PD features remain unknown, hampering the development of a successful treatment.

An *in vitro* model usually used in PD research is the neuroblastoma SH-SY5Y cell line. This line is a subline of the SK-N-SH cell line, which was established in a culture in 1970 from a bone marrow aspirate of a metastatic neuroblastoma of a four-year-old female and has undergone three rounds of clonal selection [9, 10]. The initial characterization of the SH-SY5Y cell line showed modest activity of dopamine-*β*-hydroxylase and negligible levels of choline acetyltransferase, acetylcholinesterase, butyrylcholinesterase and tyrosine hydroxylase activities, and basal noradrenaline release [11]. In order to create SH-SY5Y-derived cell models that mimic PD, we choose the pharmacological strategy (6-hydroxydopamine (6-OHDA) treatment). 6-Hydroxydopamine is a structural analogue of catecholamines, dopamine, and noradrenaline and exerts toxic effects on catecholaminergic neurons [12]. Although the 6-OHDA model does not cover all PD symptoms, it does reproduce the main cellular processes involved in PD. Up to now, four main mechanisms have been proposed to explain the cytotoxic action of 6-OHDA: (1) extra- or intracellular autooxidation of 6-OHDA proceeding with the formation of superoxide, hydrogen peroxide (H_2_O_2_), and hydroxyl radicals [2, 13]; (2) enzymatic formation of H_2_O_2_ due mainly to the action of monoamine oxidase [3, 14]; (3) direct inhibition of mitochondrial respiratory chain complex I [4, 15]; and (4) dysregulation of autophagy [5, 16]. These mechanisms may act independently or in combination to generate reactive oxygen species (ROS) [17]. The resulting oxidative stress (OS) may be intensified by an augmentation of cytoplasmic free calcium ions (as a result of either glutamate excitotoxicity or increase in mitochondrial membrane permeability), finally inducing cell death [18]. Upon import by dopamine active transporters in dopamine neurons [19], 6-OHDA was reported to undergo rapid autooxidation (~15 min) producing hydrogen peroxide, hydroxyl radicals, and peroxynitrite and inducing a transient increase in the mitochondrial superoxide level [20, 21]. Furthermore, 6-OHDA elicits dynamin-related protein-1- (Drp1-) dependent mitochondrial fission and loss of mitochondrial content by upregulating Beclin-1-independent mitophagy and alters microtubule dynamics [22]. 6-Hydroxydopamine was reported to cause global repression of prosurvival transcription programs including decreased cyclic AMP-regulated protein kinase signaling, to promote the mislocalization of prosurvival transcription factors from the nucleus to the cytosol [23] and to block nuclear import of prosurvival transcription factors in SH-SY5Y cells [24–26].

**Figure 1:**
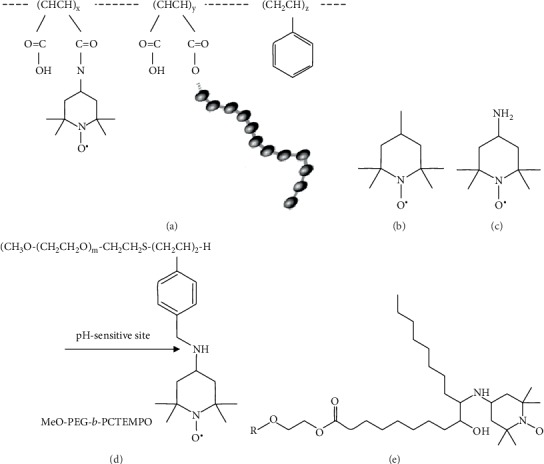
Structures of studied antioxidant compounds: nitroxide radical-containing redox nanoparticles 1 (copolymer based on poly(styrene-co-malein anhydride; NRNP1) (a), free nitroxide radicals: 2,2,6,6-tetramethylpiperidine-1-oxyl (TEMPO) (b), 4-amino-2,2,6,6-tetramethylpiperidine-1-oxyl (4-amino-TEMPO) (c), pH-sensitive radical-containing nanoparticles (NRNP2) (d), and nitroxide radicals-containing redox nanoparticles 3 (a copolymer of sorbitan-fatty acid esters with 4-amino-TEMPO; NRNP3) (e).

Antioxidants have been proposed to ameliorate the development of neurodegenerative diseases including PD [27]. Nevertheless, the effects of antioxidant intervention, including increased consumption of dietary antioxidants, were reported to be generally modest [28, 29]. These findings suggest that new antioxidant compounds, of increased efficacy, should be searched for. Particularly, synthetic antioxidants seem more promising in this respect as they allow combining different functions within one molecule and overcoming limitations of evolutionary selected biosynthetic pathways. Apart from the antioxidant function, these compounds may have other beneficial actions; e.g., they can interfere with protein aggregate formation or inhibit undesired enzymatic activities. Free nitroxyl radicals (nitroxides), such as 4-hydroxy-2,2,6,6-tetramethylpiperidine-1-oxyl (TEMPOL), are promising in this respect [30]. They are many-faceted antioxidants, which have enzyme-like, catalytic superoxide dismutase activity; inhibit the Fenton reaction by the ability to oxidize transition metal ions; and terminate radical chain reactions by radical recombination. They can accept electrons from the mitochondrial electron transport chain. Moreover, they react also with protein tyrosyl and tryptophanyl radicals [31]. We have demonstrated that nitroxides are efficient, i.e., in preventing glycation and reactions of peroxynitrite [32, 33]. A drawback of nitroxides is their short life time *in vivo* and toxic effects, including induction of apoptotic cell death at high concentrations [34, 35] and lowering of blood pressure *in vivo* [36]. Lately, drug delivery systems using nanomedicines have been proposed; they are expected to have various clinical applications [37]. For example, since nanoparticles with long-term blood circulation specifically accumulate in tumour tissues after intravenous administration [38], controlled drug release from nanoparticles at tumour sites increases the therapeutic effects of the anticancer drug and suppresses its severe side effects [39]. It can be expected that the application of a nanotechnological solution consisting of nanoparticles containing covalently bound nitroxides (nitroxide radical-containing redox nanoparticles (NRNPs)) to neurons will be much more efficient than treatment with natural antioxidants. This redox polymer contains nitroxide radicals as ROS scavengers in the hydrophobic segments bound via covalent linkages and forms a polymeric micelle of about 40 nm diameter under physiological conditions, which confines the nitroxide radicals in its core [40]. The main aim of our studies was the evaluation of NRNP1 (copolymer based on poly(styrene-co-malein anhydride) (Figure 1(a))) [41] toxicity and its ability to penetrate the blood-brain barrier (BBB) as well as its efficacy in ameliorating 6-OHDA toxicity in the cellular SH-SY5Y model of PD, in comparison with free nitroxides (2,2,6,6-tetramethylpiperidine-1-oxyl (TEMPO; Figure 1(b)) or 4-amino-2,2,6,6-tetramethylpiperidine-1-oxyl (4-amino-TEMPO; Figure 1(c))). In initial experiments, a pH-sensitive radical-containing nanoparticle (designed and developed using a self-assembling amphiphilic block copolymer (PEG-b-PCTEMPO)) composed of a hydrophilic poly(ethylene glycol) (PEG) segment and a hydrophobic poly(chloromethylstyrene) (PCMS) segment in which the chloromethyl groups were converted to 2,2,6,6-tetramethylpiperidinyloxys (TEMPOs) via the amination of PEG-b-PCMS block copolymer with 4-amino-TEMPO (NRNP2; Figure 1(d)), and a copolymer of sorbitan-fatty acid esters with 4-amino-TEMPO (NRNP3; Figure 1(e)) were also used.

## 2. Materials and Methods

### 2.1. Materials and Equipment Used in Studies of Cell-Free Systems

Xylenol orange (cat. no. chem^∗^237045902^∗^5 g) was obtained from Polish Chemical Reagents (POCH, Gliwice, Poland); fluorescein (cat. no. chem^∗^114277705^∗^10 g), perchloric acid (HClO_4_; cat. no. chem^∗^115649402^∗^1 l), and sodium hypochlorite (NaOCl, 15% active chlorine basis, cat. no. 528066510) were purchased from Chempur (Piekary Śląskie, Poland); phosphate-buffered saline (PBS: 145 mM NaCl, 1.9 mM NaH_2_PO_4_, and 8.1 mM Na_2_HPO_4_, cat. no. PBS405) was obtained from Lab Empire (Rzeszów, Poland), and DMEM/F12 (cat. no. 11039-021) was purchased from Thermo Fisher Scientific (Waltham, MA, USA). 5-Amino-3-(4-morpholinyl)-1,2,3-oxadiazolium chloride (SIN-1 chloride, OONO^−^ donor, cat. no. 0756/50) was obtained from Tocris Bioscience (Bristol, United Kingdom). SIN-1 chloride solutions (1 mM) were prepared in PBS and aliquots were frozen and kept at −80°C until use. HPLC analysis proved that under these conditions, SIN-1 chloride was stable for several months. 2,2-Azobis(2-amidinopropane) dihydrochloride (AAPH, cat. no. 440914) was purchased from Polysciences (Warrington, PA, USA). A stock solution of AAPH was freshly prepared in PBS before each experiment, kept on ice, and used daily. Dihydrorhodamine 123 (DHR123) (cat. no. D23806) was purchased from Thermo Fisher Scientific (Waltham, MA, USA). A stock solution of NaOCl was diluted in 0.1 M NaOH, and its concentration was determined spectrophotometrically at 290 nm using the molar absorption coefficient of *ɛ*_290 nm_ = 350 M^−1^ cm^−1^ [42]. Under such conditions NaOCl exists exclusively as OCl^−^. Before use, the stock solution of NaOCl was diluted in PBS. At pH 7.4, both forms, HOCl and OCl^−^, are present in the solution at comparable concentrations. All other reagents, if not mentioned otherwise, were purchased from Sigma-Aldrich Corp. (St. Louis, MO, USA) and were of analytical grade. 6-Hydroxydopamine hydrobromide (6-OHDA) (cat. no. H116) was provided by Sigma-Aldrich (St. Louis, MO, USA). Distilled water was purified using a Milli-Q system (Millipore, Bedford, MA, USA). Fluorometric and absorptiometric measurements were done in a Tecan Infinite 200 PRO multimode reader or a Spark multimode microplate reader (Tecan Group Ltd., Männedorf, Switzerland). All measurements were performed in triplicate and repeated at least three times on different preparations.

### 2.2. Materials and Equipment Used to Study the Neuroblastoma Cell Line

The certified human neuroblastoma cell line SH-SY5Y (ATCC CRL-2266) was purchased from American Type Culture Collection (ATCC, Rockville, MD, USA). Dulbecco's Modified Eagle Medium Nutrient Mixture F-12 (DMEM/F12) without Phenol Red (cat. no. 11039-021), Dulbecco's phosphate-buffered saline 1x with Ca^2+^ and Mg^2+^ ions, Geltrex™ LDEV-Free Reduced Growth Factor Basement Membrane Matrix (cat. no. A1413202), and MitoTracker Deep Red FM (cat. no. M22426) were obtained from Thermo Fisher Scientific (Waltham, MA, USA). Foetal bovine serum (FBS, cat. no. 04-001-1A), 10x Trypsin-EDTA solution (cat. no. 03-051-5B), PBS without Ca^2+^ and Mg^2+^ ions (cat. no. 02-023-1A), and Penicillin-Streptomycin Solution (cat. no. 03-031-1B) were obtained from Biological Industries (Cromwell, CT, USA). The tetrazolium dye 3-(4,5-dimethylthiazol-2-yl)-2,5-diphenyltetrazolium bromide (MTT, cat. no. M2128), 2,2,6,6-tetramethylpiperidine-1-oxyl (TEMPO, cat. no. T-7263), 4-amino-2,2,6,6-tetramethylpiperidin-1-yloxyl (4-amino-TEMPO, cat. no. 163945), 0.4% Trypan Blue solution (cat. no. T8154), isopropanol (cat. no. I9516), *N*-ethylmaleimide (NEM, cat. no. E3876), trichloroacetic acid (TCA, cat. no. T4885), diethylenetriaminepentaacetic acid (DTPA, cat. no. D1133), L-ascorbic acid (cat. no. A0278), *ortho*-phthalaldehyde (cat. no. P1378), L-glutathione reduced (GSH, cat. no. PHR1359), dihydroethidium (DHE, cat. no. D7008), 4′,6-diamidino-2-phenylindole (DAPI, 4′,6-diamidino-2-phenylindole, dihydrochloride) (cat. no. D9542), Triton X-100 (cat. no. 9002-93-1), phalloidin conjugated with Atto-488 (cat. no. 49409), and acridine orange 10-nonyl bromide (NAO, cat. no. A7847) were provided by Sigma-Aldrich (St. Louis, MO, USA). 96% ethanol (cat. no. 396420113), 37% Formaldehyde Solution, and hydrochloric acid 35-38% (cat. no. 115752837) were provided by Chempur (Poland). CellTiter-Glo® Luminescent Cell Viability Assay (cat. no. G7571) was purchased from Promega (Madison, WI, USA). JC-1 Mitochondrial Membrane Potential Assay Kit was purchased from Abnova (Taiwan, China). Cell Meter™ Fluorimetric Mitochondrial Superoxide Activity Assay Kit Optimized for Microplate Reader was provided by AAT Bioquest (Sunnyvale, CA, USA).

Cell culture 75 cm^2^ flasks (cat. no. 156499), transparent 96-well culture plates (cat. no. 161093), and black (cat. no. 165305) and white (cat. no. 165306) 96-well plates with an optical bottom were obtained from Thermo Fisher Scientific (Waltham, MA, USA). Other sterile cell culture materials and 24-well plates were purchased from Nerbe (Winsen, Germany) and NEST Biotechnology (Wuxi, China).

6-Hydroxydopamine hydrobromide stabilized with 0.01% ascorbic acid was reconstituted according to the manufacturer's protocol in 2 mL of PBS, aliquoted; 10 mM stocks were frozen in -20°C; 4-amino-TEMPO (10 mM stock solution) was dissolved in PBS and both of them were filtered using 0.22 *μ*m filter. TEMPO (50 mM stock solution) was dissolved in dimethyl sulfoxide (DMSO); the final highest concentration of DMSO in cell media was ≤0.02%.

The radical-containing nanoparticles used for the initial experiments were synthesized by the group of Prof. Nagasaki (University of Tsukuba) (NRNP1: 1.64 mM stock solution in water; the pH-sensitive radical-containing-nanoparticle, NRNP2: 1.81 mM stock solution in water) [41, 43]. Then, syntheses of the NRNPs (NRNP1, NRNP2, and NRNP3) were conducted based on the experience of the team of Dr. J. Skolimowski (Department of Analytical Biochemistry, University of Rzeszow). Electron paramagnetic resonance signals were measured to estimate the amount of nitroxyl radicals in the nanoparticles.

Distilled water was purified using a Milli-Q system (Millipore, Bedford, MA, USA). Fluorometric and absorptiometric measurements were done using a Spark multimode microplate reader (Tecan Group Ltd., Männedorf, Switzerland). Images were taken using a ZEISS LSM 710 inverted confocal microscope (Oberkochen, Germany). All measurements were performed at least in triplicate (usually ninefold) and repeated at least three times on different preparations.

### 2.3. Materials Used to Study the Human Immortalized Brain Endothelial Cell Line

The *in vitro* studies were carried out also on the human immortalized brain endothelial cell line (hCMEC/D3) (kindly donated by Prof. Pierre Couraud from INSERM, Paris, France [44]). Rat collagen (cat. no. I #3440-100-01) by Cultrex was obtained from R&D Systems, (McKinley Pl NE, MN, USA). Human basic fibroblast growth factor (bFGF; cat. no. F0291-25UG), L-ascorbic acid (cat. no. A4544), HEPES Buffer (cat. no. 1 M–HO887), and hydrocortisone (cat. no. H-0135; 1 mg) were purchased from Sigma-Aldrich, (St. Louis, MO, USA). Chemically Defined Lipid Concentrate (cat. no. 11905-031) and Pen-Strep (cat. no. 15140122) were provided by Gibco (Thermo Fisher Scientific, Waltham, MA, USA). Trypsin 0.05%/EDTA, 0.02% in PBS, without Ca^2+^ and Mg^2+^ (cat. no. P10-023100) was purchased from PAN-Biotech GmbH (Aidenbach, Germany).

Endothelial cell growth basal medium-2 (EBM-2) (cat. no. CC3156) was obtained from Lonza (Basel, Switzerland); foetal bovine serum (FBS Superior; cat. no. S 0615) was provided by Biochrom (Merck, Germany). Thin Cert–Tissue Culture Inserts for Multiwell Plates (cat. no. 662640) were obtained from Greiner Bio-One (Kremsmünster, Austria).

### 2.4. NRNP Characterization

#### 2.4.1. Scanning Electron Microscopy

The morphology of studied NRNPs was visualized using scanning electron microscope (SEM) with energy-dispersive X-ray spectroscopy (EDS) analyzer Quanta™ 3D 200i (FEI Co. Field Emission Instruments, Hillsboro, OR, USA).

#### 2.4.2. Electron Spin Resonance (ESR) Spectroscopy

Nitroxide residues per sum of hydrophilic and hydrophobic segment masses (molecular weight) were determined using ESR. Nitroxide signal intensity was measured immediately after addition of selected concentration of NRNPs or free nitroxides using microhematocrit capillaries (nonheparinized microhematocrit tubes ∼75 *μ*L; 1.55 × 75 mm; Medlab Products, Raszyn, Poland) in a Bruker multifrequency and multiresonance FT-EPR ELEXSYS E580 apparatus (Bruker BioSpin, Billerica, MA, USA). The spectrometer operated at X-band (9.850537 GHz). The following settings were used: central field, 3353.0 G; modulation amplitude, 1 G; modulation frequency, 100 kHz; microwave power, 23.77 mW; power attenuation 8.0 dB; scan range, 100 G; conversion time, 25 ms; and sweep time, 25.6 s. The spectra were recorded in 1024 channels, with number of scans, 3. The spectra were recorded and analysed using Xepr 2.6b.74 software. Xepr is a comprehensive software package of the ELEXSYS series, accommodating the needs of every user with highly developed acquisition and processing tools. The signal was integrated twice to determine its area and thus the concentration of the radical.

#### 2.4.3. Penetration of NRNPs into SH-SY5Y Cells

Cells from about 80% confluent T-75 flask were trypsinized, centrifuged (5 min, 900 rpm) and resuspended in 3 mL of medium containing 100 *μ*M NRNP1; divided into three parts; and incubated for 1, 2, and 4 hours, respectively. After an appropriate period of time, cells were centrifuged, and the pellet was washed with PBS and then centrifuged once again (supernatant was collected for further examination). The pellet was suspended in 200 *μ*L PBS. Signal intensity of the nitroxide was measured using microhematocrit capillaries.

### 2.5. Analysis of Cell-Free Systems

#### 2.5.1. Antiradical Activity: ABTS^∗^ Scavenging

The antioxidant properties of 6-OHDA were estimated using the 2,2′-azinobis(3-ethylbenzthiazoline-6-sulfonic acid) radical (ABTS^∗^) according to a procedure previously proposed by us [45]. Appropriate amounts of 6-OHDA solution and of solutions of selected amino acids were added to a solution of ABTS^∗^and diluted so that 200 *μ*L of the solution had an absorbance of 1.0 in a microplate well, at 734 nm. The decrease in ABTS^∗^ absorbance was measured after 1 min (“fast” scavenging) and between 10 and 30 min (“slow” scavenging) of incubation at room temperature (21 ± 1°C). From the plots of the dependence of absorbance decrease (*Δ*A) on the compound concentration, the value of *Δ*A/mM was calculated for the compounds tested based on a Trolox calibration curve. The compounds studied were dissolved in PBS.

#### 2.5.2. Assay of Hydrogen Peroxide Generation

Evaluation of H_2_O_2_ generation by 100 *μ*M (final concentration) 6-OHDA solution in DMEM/F12 complete medium without phenol red was performed by incubation of the samples for 3 h at 37 ± 1°C with shaking. The peroxide content was estimated before and after incubation by the ferric-xylenol orange method [46]. After that, 180 *μ*L/well of samples and 20 *μ*L/well of the xylenol orange reagent were mixed together (2.5 mM xylenol orange/2.5 mM Mohr's salt (Fe (NH_4_)_2_(SO_4_)_2_) in 1.1 M perchloric acid). The absorbance was measured at 560 nm after a 30-minute incubation at room temperature.

#### 2.5.3. Protection against Oxidation of Dihydrorhodamine 123 (DHR123)

Dihydrorhodamine 123 (190 *μ*L of 1 *μ*M solution in 0.1 M phosphate buffer, pH 7.4) was added to each well of a 96-well plate containing the compounds studied in a range of concentrations (0.005–10 *μ*M). The final volume of a sample was 200 *μ*L. SIN-1 (1 *μ*L of 1 mM solution) was added to each well, and kinetic measurement of fluorescence increase was carried using the excitation/emission wavelengths of 460/528 nm at 37°C for 2 h. From the area under curve values of fluorescence, IC_50_ values were determined.

#### 2.5.4. Protection of Fluorescein against Bleaching Induced by NaOCl or AAPH

Inhibition of fluorescein bleaching was determined with a method proposed by us [45]. Briefly, an aliquot of hypochlorite was added to a microplate well containing 100 *μ*L of 0.2 *μ*M fluorescein dissolved in PBS and the solution was mixed immediately. The amount of hypochlorite required to decrease fluorescence down to ca 5–10% of the initial value was determined (it corresponded to 1.75 nmol of hypochlorite). These conditions were used for subsequent measurements, in which nitroxides and NRNPs dissolved in PBS in a range of concentrations (0.125–10 *μ*M) were added to the fluorescein solution before addition of hypochlorite, keeping the volume of the sample constant (100 *μ*L). Fluorescence was measured after 15 min incubation at room temperature at the excitation/emission wavelengths of 485 and 538 nm, respectively.

AAPH stock solution was added to wells containing 0.2 *μ*M fluorescein dissolved in PBS to obtain the final concentration of 10 mM and the solution was mixed immediately. These conditions were used for subsequent measurements, in which compounds dissolved in PBS in a range of concentrations (0.25–10 *μ*M) were present in the fluorescein solution before the addition of AAPH, keeping the volume of the sample constant (100 *μ*L). Fluorescence was measured after 1 hour incubation at 37°C. Percent of protection was calculated according to the formula:

(1) $$\begin{array}{c} \%\text{ Protection}=\left({\text{F}}_{\text{n}}‐{\text{F}}_{\text{o}}\right)/\left({\text{F}}_{\text{c}}‐{\text{F}}_{\text{o}}\right)\times 100\% \end{array}$$

where *F*_n_ is fluorescence of a sample containing fluorescein, hypochlorite/AAPH, and a compound studied; *F*_o_ is fluorescence of fluorescein treated with hypochlorite/AAPH only; and *F*_c_ is fluorescence of the nontreated fluorescein.

From the concentration dependence of protection on the antioxidant concentration, the concentrations of compounds providing 50% protection (IC_50_) against the fluorescein bleaching were calculated.

### 2.6. Assessment of Penetration across a Model of the Blood-Brain Barrier (BBB) by NRNP1 and Free Nitroxides in an *In Vitro* Model

#### 2.6.1. hCMEC/D3 Cell Culture

The human immortalized brain endothelial line cells (hCMEC/D3) [44] were seeded onto culture flasks previously coated with collagen I (150 *μ*g/mL, at 37°C for a least one hour) and maintained in endothelial cell basal medium 2 (EBM-2) containing 5% FBS. 1% penicillin-streptomycin, hydrocortisone (1.4 *μ*M), ascorbic acid (5 *μ*g/mL), 1% chemically defined lipid concentrate, and HEPES (10 mM), bFGF (1 ng/mL) under standard conditions: 37°C, 100% humidity, and the atmosphere being 5% CO_2_ and 95% air. Experiments were performed on cells from the passages between 26 and 35. The cells were periodically tested for Mycoplasma.

#### 2.6.2. Transport across a Model of the Blood-Brain Barrier

The transport of NRNP1 as well as free nitroxides across BBB was analysed using transwell inserts (ThinCert™, Greiner Bio-One). The hCMEC/D3 cells were plated onto sterile 24-well cell culture inserts (pore diameter 0.4 *μ*m), coated with collagen I, and grown to confluence for approximately five days. For the experiment, a medium with a final concentration (5 *μ*M) of compounds studied was prepared directly before adding. The culture medium was replaced in either top or bottom compartment by a medium with appropriate compounds, and after 1, 20, 40, or 60 min, inserts were removed from lower reservoirs and solutions were transferred from lower reservoirs and from inserts to a fresh 96-well plate. Collected samples were measured three times in a Bruker multifrequency and multiresonance FT-EPR ELEXSYS E580 apparatus. Before the experiment, the transendothelial electrical resistance (TEER) was measured in duplicate inserts (five times in every single insert) using the epithelial volt-ohm meter Millicell® ERS-2 (Millipore) with MERSSTX01 electrode. All TEER values were determined after subtracting the background (TEER for cells free insert coated with collagen I) and by correction for surface area. The values were >40 *Ω* cm^−2^. Wells showing too low TEER values were eliminated from the measurements [47].

#### 2.6.3. Electron Spin Resonance (ESR) Spectroscopy

ESR signal intensity of free nitroxides of NRNPs (∼15 *μ*L) was measured using microhematocrit capillaries (nonheparinized microhematocrit tubes; 1.55 × 75 mm; Medlab Products, Raszyn, Poland) in a Bruker multifrequency and multiresonance FT-EPR ELEXSYS E580 apparatus (Bruker BioSpin, Billerica, MA, USA). The spectrometer was operated at X-band (around 9.4 GHz). The following settings were used: central field, around 3354.0 G; modulation amplitude, 0.3 G; modulation frequency, 100 kHz; microwave power, 94.64 mW; power attenuation 2.0 dB; scan range, 100 G; conversion time, 25 ms; and sweep time, 25.6 s. The spectra were recorded with 1024 points per scan, with the accumulated number of scans, 3. The spectra were recorded and analysed using Xepr 2.6b.74 software. The signal was integrated twice to determine its area and thus the concentration of the radical.

### 2.7. SH-SY5Y Cell Culture

SH-SY5Y cell line was cultured in DMEM/F12 without phenol red, supplemented with 10% *v*/*v* heat-inactivated foetal bovine serum (hi-FBS) and 1% *v*/*v* penicillin and streptomycin solution. Cells were maintained at 37°C in 5% carbon dioxide and 95% humidity. The medium was changed twice a week, and the cells were passaged at about 80% confluence. For all studies, cells up to 14 passages were used. The morphology was examined under an inverted microscope with phase contrast Zeiss Primo Vert (Oberkochen, Germany); cell viability was estimated by Trypan Blue exclusion test and cells were counted using Thoma hemocytometer (Marienfeld Superior, Lauda-Königshofen, Germany).

Optimal cell number was experimentally determined. Cells were seeded into covered plates at various densities 2, 3, 3.5, 4, and 5 × 10^4^ cells/well of a 96-well plate in 100 *μ*L of cell culture medium and incubated for 24 hours. Then, a MTT assay was performed as described below. Another cytotoxicity assay, using Neutral Red has proved to be unsuitable due to significant cell loss during multiple washing steps.

#### 2.7.1. Cell Viability Assay

Human neuroblastoma cells were seeded in 96-well clear plate previously covered with 1% Geltrex™ LDEV-Free Reduced Growth Factor Basement Membrane Matrix (according to the manufacturer's protocol) at an amount of 3.5 × 10^4^ cells/well in 100 *μ*L culture medium (optimal cell number determined as described above). After 24 hour incubation, the medium was gently removed and replaced with cell culture medium supplemented with adequate compounds (6-OHDA, redox nanoparticles (NRNP1, NRNP2, and NRNP3), 4-amino-TEMPO, or TEMPO) at various concentrations in order to evaluate their self-cytotoxicity. After 24-hour exposure, the medium was removed and replaced with 100 *μ*L of 0.5 mg/mL MTT solution in 1x PBS with ions and incubated for 4 hours in a CO_2_ incubator. Then, 100 *μ*L/well of isopropanol : HCl (250: 1 *v*/*v*) solution was added into cells in order to dissolve formazan crystals and shaken for about 20-30 minutes. The dissolution was controlled under inverted microscope. Absorbance was measured at 570 nm.

#### 2.7.2. Treatment of SH-SY5Y Cells with NRNPs and Free Nitroxides

In order to analyse antineurodegenerative properties of the compounds studied (NRNP1 and free nitroxides), cells were seeded as described above (SH-SY5Y Cell Culture and Cell Viability Assay). After overnight incubation to allow cell adhesion, the medium was replaced with 50 *μ*L/well of the studied compounds in the medium at chosen concentrations (4-amino-TEMPO and NRNP1: 75, 100, and 150 *μ*M or TEMPO: 100, 150 *μ*M). Subsequent to 2-hour preincubation with the antioxidants, 50 *μ*L/well of 120 *μ*M 6-OHDA was added (final concentration: 60 *μ*M, slightly lower than IC_50_) and then incubated, respectively, for 1 or 24 hours.

#### 2.7.3. Measurement of Reduced Glutathione Content

The content of reduced glutathione (GSH) was assayed with *ortho-*phthalaldehyde (OPA) [48]. SH-SY5Y cells were seeded in a clear 96-well plate at an amount of 4 × 10^4^ cells/well in 100 *μ*L culture medium and treated with selected antioxidants as described in Section 2.7.2. GSH was measured after 1 and 24 h incubation with 60 *μ*M 6-OHDA. Following incubation with 6-OHDA, the medium was gently removed and cells were washed with PBS (150 *μ*L/well). Phosphate-buffered saline was gently removed by aspiration. Subsequently, 60 *μ*L/well of freshly prepared cold lysis buffer (RQB buffer: 20 mM HCl, 5% TCA, 5 mM DTPA, and 10 mM L-ascorbic acid) was added; then, the plates were shaken at 900 rpm for 5 minutes and centrifuged at 4000 rpm (5 minutes, room temperature).

Cell lysates were transferred into two separate black 96-well plates with a black bottom in a volume of 25 *μ*L/well afterwards (‘+ NEM' and ‘- NEM'). Into the first plate ‘+ NEM', 4 *μ*L/well of newly prepared 7.5 mM NEM in ice-cold RQB buffer were added. Then, 40 *μ*L/well of 1 M phosphate buffer (pH 7.0) was pipetted into both plates, which were shaken for 5 minutes at 900 rpm. Then, 160 *μ*L/well of ice-cold 0.1 M phosphate buffer (pH 6.8) and 25 *μ*L/well of newly prepared 0.5% OPA in methanol were added into ‘+NEM' and ‘-NEM' plates. Then, the plates were shaken at 900 rpm for 30 minutes. Fluorescence was measured with a TECAN Spark® multimode plate reader at 355/430 nm. GSH concentration was established by subtracting the fluorescence of the plate without NEM from the fluorescence of the NEM-containing plate and GSH content was calculated, respectively, with reference to protein content in each well.

#### 2.7.4. Protein Assay

Protein content was determined according to Lowry et al. [49].

#### 2.7.5. Assessment of Intracellular ATP Level

Intracellular ATP level was determined using CellTiter-Glo® Luminescent Cell Viability Assay (Promega, Madison, WI, USA), which is based on the enzymatic and luminescent transformation of luciferin to oxyluciferin in the presence of ATP. Cells were seeded into white 96-well plate with an optical bottom, cultured and treated with adequate compounds as described in Sections 2.7.1 and 2.7.2. Cells were assayed after 1 and 24 h incubation in separate plates (assessing both short- and long-term effects of 6-OHDA), by adding 100 *μ*L of CellTiter-Glo® Reagent to the cell culture medium present in each well, shaken and incubated according to the manufacturer's protocol. Luminescence was recorded using a TECAN Spark® multimode plate reader (Tecan Group Ltd., Switzerland).

#### 2.7.6. Measurement of Reactive Oxygen Species (ROS) Using Fluorescent Probe (DHE)

Cells were seeded, cultured, and posttreatment handled as previously described (Section 2.7.2) onto black 96-well plates with a clear bottom. This test was performed after 1 h or 24 h incubation with 6-OHDA of cells preincubated with antioxidants. 100 *μ*L/well of freshly prepared DHE working solution in PBS was added; the final concentration of DHE is equal to 10 *μ*M. The fluorescence was measured immediately at 37°C, at 405/570 nm for 2 hours, and at 1 min intervals.

#### 2.7.7. Fluorimetric Estimation of Mitochondrial Superoxide Radical Level (O_2_^·–^)

Mitochondrial superoxide level was measured using Cell Meter™ Fluorimetric Mitochondrial Superoxide Activity Assay Kit Optimized for Microplate Reader (AAT Bioquest, Sunnyvale, CA, USA). Cells were seeded, cultured, and posttreatment handled as previously described (Section 2.7.2) onto black 96-well plates with a clear bottom. A test was performed after 1 and 24 h incubation with 6-OHDA. After an appropriate period of time, assay was performed according to the manufacturer's protocol: the medium was gently removed and 100 *μ*L/well of reagent was added. The plate was incubated for 45 minutes in a CO_2_ incubator. After that, fluorescence was measured at 540/590 nm for 4 hours at 2 min intervals.

#### 2.7.8. Mitochondrial Membrane Potential Evaluation (Δ*ψ*_m_)

Mitochondrial membrane potential was assayed using JC-1 (5,5′,6,6′-tetrachloro-1,1′,3,3′-tetraethylbenzimidazolylcarbocyanine iodide) with a Mitochondrial Membrane Potential Assay Kit (Abnova, Taiwan, China). JC-1 is a dye that can selectively enter the mitochondria and reversibly shift colour from green to red as the membrane potential rises. In healthy cells with high Δ*ψ*_m_, JC-1 forms complexes well known as J-aggregates with profound red fluorescence. On the other hand, in injured cells with low Δ*ψ*_m_, JC-1 remains in the monomeric state and exhibits green fluorescence exclusively.

Cells were seeded into black microplates with an optical bottom and treated as described in Section 2.7.2. After an appropriate period of time of exposure to 60 *μ*M 6-OHDA (1 h or 24 h), the medium was softly removed and replaced with 100x diluted JC-1 reagent in complete culture medium and incubated at 37°C for 30 min. Then, a microplate was centrifuged at 4000 rpm at room temperature for 5 min. The reagent was gently removed, and cells were washed with 150 *μ*L/well Cell-Based Assay Buffer and centrifuged once again at the same conditions. Then, the buffer was removed and 100 *μ*L/well of the new buffer was added. Fluorescence was measured at 540/570 nm (red fluorescence) and 485/535 nm (green fluorescence). The results were expressed as a ratio of red to green relative fluorescence units. Mitochondrial depolarization was indicated by a decrease in the polymer/monomer fluorescence intensity ratio.

#### 2.7.9. Mitochondrial Mass Assessment

Cells were seeded at an amount of 2 × 10^5^ cells/well onto a 24-well plate and cultured as previously described (Section 2.7.2). After 24-hour exposure to 6-OHDA, cells were trypsinized, counted, transferred to Eppendorf tubes, and centrifuged for 6 minutes at 2000 rpm (the same centrifugation conditions were maintained during this assay), then washed with 1 mL of PBS and centrifuged again. Next, 1 mL of 10 *μ*M NAO (solution in PBS) was added into the samples, and cells were incubated for 10 min at 37°C. Afterwards, the cells were centrifuged and washed with 1 mL of PBS, and later, the pellet was resuspended in 300 *μ*L of PBS. Each sample was transferred into a 96-well black plate (100 *μ*L/well; 3 repetitions). Fluorescence was measured at 435/535 nm. The results were calculated with reference to the number of cells.

#### 2.7.10. MitoTracker Labeling

**Figure 2:**
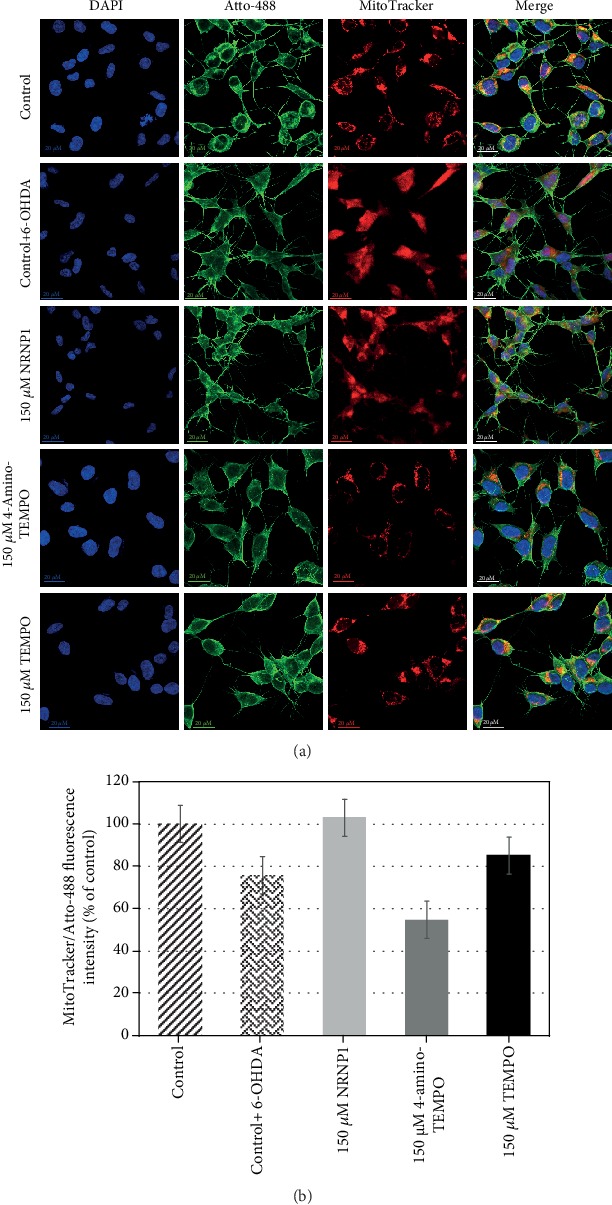
Morphological changes in the cellular structure. Confocal images of SH-SY5Y 63× preexposed to NRNP1 or free nitroxides (TEMPO or 4-amino-TEMPO) for 2 h and treated with 6-OHDA for 24 h. Nuclei were stained with DAPI (blue), phalloidin conjugated with Atto-488 (green), and mitochondria with MitoTracker Deep Red FM (red). All scale bars (control, bottom right) are 20 *μ*m (a). In ImageJ, the fluorescence intensity was expressed as a mean gray value separately for the red channel (from MitoTracker) and separately for the green channel (phalloidin). The ratio of MitoTracker to phalloidin fluorescence (i.e., the ratio of the signal from the mitochondria to the number of cells (actin skeleton)) was calculated. The calculated values were expressed as a percentage of untreated control (b).

SH-SY5Y cells were seeded on a 8-well chamber slide (Lab-Tek™ II Chamber Slide™ System cat. no. 154534, Thermo Scientific, Waltham, MA, USA) at an amount of 7 × 10^4^ cells/400 *μ*L culture medium and then treated as described in Section 2.7.2 long treatment). Following the treatment, the medium was removed and replaced with 250 nM solution of MitoTracker Deep Red FM (cat. no. M22426, Thermo Scientific, Waltham, MA, USA) in PBS and incubated for 45 min, in CO_2_ incubator. Then, cells were washed with PBS (200 *μ*L/well) and fixed with 200 *μ*L/well 3.7% formaldehyde for 15 min. After that, cells were washed with PBS (200 *μ*L/well), permeabilized with 0.1% Triton X-100 solution at an amount of 200 *μ*L/well for 10 min, and washed with PBS (700 *μ*L/well). Then, 150 *μ*L/well of phalloidin working solution (prepared accordingly to the manufacturer's protocol) was added and for 60 min incubated for 60 min. After washing the cells with PBS (700 *μ*L/well), nuclei were stained with 600 nM DAPI (200 *μ*L/well). Images were taken using a Zeiss LSM 710 inverted confocal microscope (Oberkochen, Germany) under 63x magnification. The ratio of fluorescence intensity of the mitochondria and cytoskeleton (expressed as mean gray value) was calculated using ImageJ software and results were graphed for the images in Figure 2(a).

### 2.8. Statistical Analysis

Kruskal–Wallis test or Student *t*-test was performed to estimate the differences between 6-OHDA-treated control cells and antioxidant-treated cells to assess their properties in each individual assay; *P* ≤ 0.05 was considered statistically significant in both cases. Also, differences between the 6-OHDA-treated and nontreated controls were assessed by an appropriate test (one of those described above). Statistical analysis of the data was performed using STATISTICA software package (version 13.3, StatSoft Inc. 2016, Tulsa, OK, USA, http://www.statsoft.com).

## 3. Results

### 3.1. Analysis of NRNPs in Cell-Free Systems

#### 3.1.1. Scanning Electron Microscopy (SEM)

**Figure 3:**
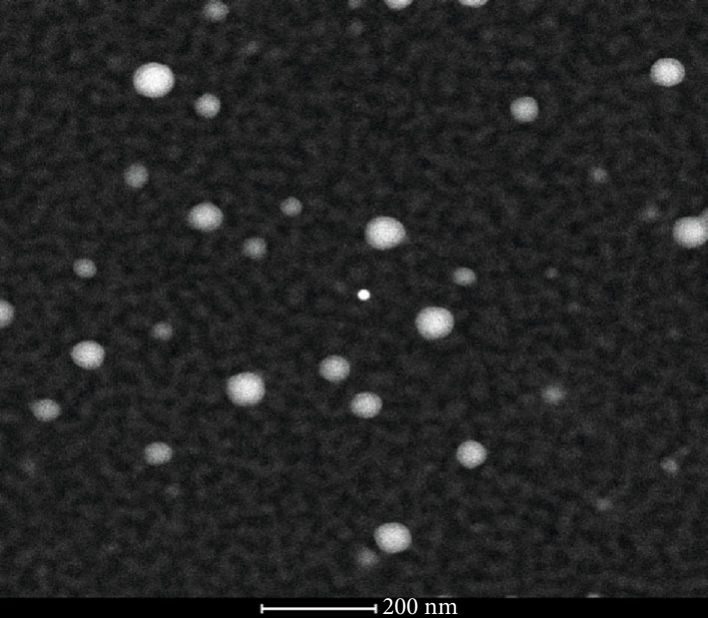
SEM picture of NRNP1.

In the scanning electron microscope, NRNP1 were visible as spherical structures of diameter of up to 100 nm (mean diameter of about 40 nm) (Figure 3).

#### 3.1.2. Content of Nitroxyl Residues in the Polymers

Nitroxide residues per sum of hydrophilic and hydrophobic segment masses (segment molecular weight) were determined using ESR and 4-amino-TEMPO as a standard of ESR signal. The results indicated 27.6 radical residues per molecular weight of a segment (7500 g/mol) for NRNP1, 7.7 radical residues per segment molecular weight (8300 g/mol) for NRNP2, and 8.4 radical residues per molecular weight of a segment of NRNP3 (2246-2417 g/mol).

#### 3.1.3. Antioxidant Properties of NRNPs

**Figure 4:**
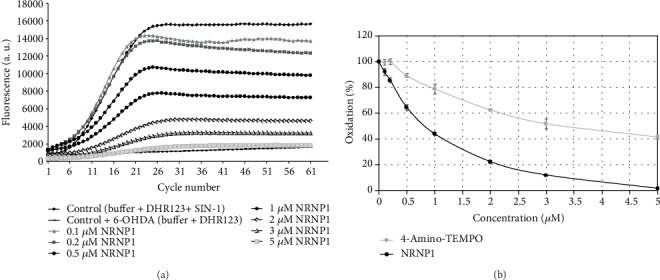
NRNP1 protect dihydrorhodamine 123 against oxidation by SIN-1: (a) kinetics of oxidation and (b) concentration dependence of the effect; for comparison, the effect of 4-amino-TEMPO is shown.

NRNPs dose dependently inhibited DHR123 oxidation induced by SIN-1 chloride (Figure 4) and fluorescein bleaching induced by NaOCl and AAPH. The nanoparticles were more effective than 4-amino-TEMPO as reflected by lower IC_50_ values (Table 1). These results demonstrate good antioxidant properties of NRNPs.

#### 3.1.4. Behaviour of 6-Hydroxydopamine in Cell-Free Systems

No production of ROS was found upon incubation of 6-OHDA in DMEM/F12 using H_2_DCFDA and DHE (not shown). Nevertheless, small amounts of H_2_O_2_ were generated (much lower than in the case of ascorbate). The commercial preparation of 6-OHDA used is stabilized with ascorbate (ca 568 *μ*M ascorbate/10 mM 6-OHDA). If the H_2_O_2_ produced was due to autoxidation of ascorbate, up to 6 *μ*M H_2_O_2_ would be generated by autoxidation of 100 *μ*M 6-OHDA solution; the value found indicates autoxidation of 6-OHDA in the cell culture medium leading to the production of H_2_O_2_. However, 6-OHDA showed also some antioxidant properties, being able to reduce ABTS^∗^. The “fast” scavenging (occurring within 1 min) was low (about 1/6 that of standard antioxidants, ascorbate, or glutathione) and about 1/10 of such antioxidant amino acids as tyrosine and tryptophan). Moreover, 6-OHDA showed also “slow” scavenging, determined in our assay as the reactivity between 10 and 30 min of reaction, not exhibited by other compounds studied, including ascorbate, and even higher than the “fast” scavenging (Table 2); thus, it could not be due to the stabilizing ascorbate but to the antioxidant activity of 6-OHDA itself.

### 3.2. Protective Effect of Nitroxide Redox Nanoparticles on SH-SY5Y Cells Treated with 6-Hydroxydopamine

#### 3.2.1. Protection of SH-SY5Y Cells against the Cytotoxicity of 6-OHDA

**Figure 5:**
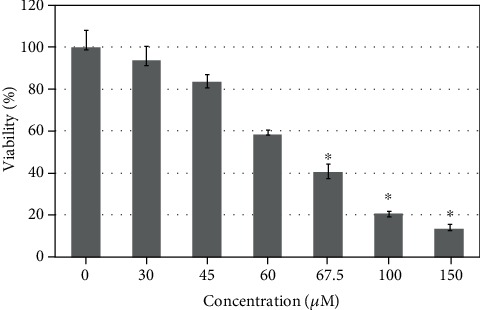
Cytotoxicity of 6-hydroxydopamine after 24 hour treatment. The whiskers are in lower (25%) and upper (75%) quartile ranges. ^∗^*P* ≤ 0.05, Kruskal–Wallis test against the nontreated control; *n* = 9.

**Figure 6:**
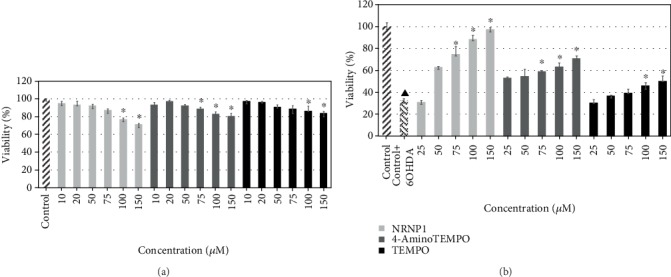
Self-cytotoxicity of selected antioxidants after 24 hour treatment (a) and their protective properties against the cytotoxicity of 60 *μ*M 6-hydroxydopamine (b). The whiskers are in lower (25%) and upper (75%) quartile ranges. ^∗^*P* ≤ 0.05, Kruskal–Wallis test against the 6-OHDA-treated control. ^▲^*P* ≤ 0.05 differences between the controls; *n* = 9.

**Figure 7:**
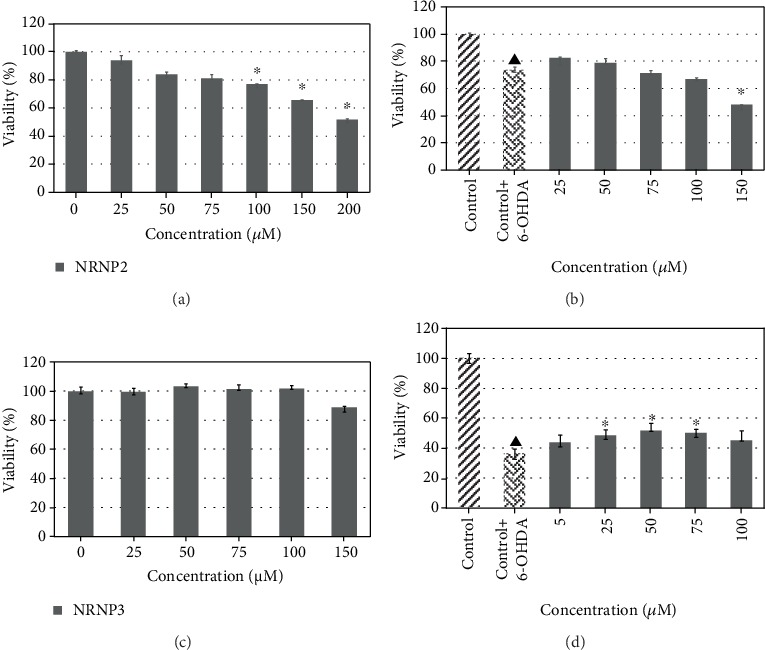
Self-cytotoxicity of NRNP2 after 24 hour treatment (a) and their protective properties of NRNP2 against the cytotoxicity of 60 *μ*M 6-hydroxydopamine (b) and self-cytotoxicity of NRNP3 after 24 hour treatment (c) and protective properties of NRNP3 against the cytotoxicity of 60 *μ*M 6-hydroxydopamine (d). The whiskers are in lower (25%) and upper (75%) quartile ranges. ^∗^*P* ≤ 0.05, Kruskal–Wallis test against the nontreated control. ^▲^*P* ≤ 0.05 differences between the controls; *n* = 9.

6-Hydroxydopamine showed a dose-dependent cytotoxicity against SH-SY5Y cells. The IC_50_ value of 6-OHDA is equal to 64 *μ*M after 24 h treatment (Figure 5). All antioxidants studied exhibited slight intrinsic cytotoxicity at concentrations between 75 and 150 *μ*M for 4-amino-TEMPO and NRNPs and 100 and 150 *μ*M for TEMPO, when applied alone, after 24 h incubation (Figure 6(a)). However, NRNP1 showed a profound neuroprotective effect against 6-OHDA-induced cytotoxicity at 75-150 *μ*M. The improvement in viability was about 30% and 50% better compared to 4-amino-TEMPO and TEMPO, respectively (Figure 6(b)). NRNP2 and NRNP3 offered lower protection (Figures 7(b) and 7(d)); so, they were not used in further experiments.

#### 3.2.2. Penetration of NRNP1 into SH-SY5Y Cells

After 1 h, 2 h, and 4 h incubation of 100 *μ*L of 100 *μ*M NRNP1 with 3 × 10^6^ cells, about 1.84%, 34%, and 32% of the nanoparticles, respectively, was internalized by the cells (judging from comparison of intensities of EPR signal after oxidation of the samples with 1 mM K_3_[Fe (CN)_6_]).

#### 3.2.3. Penetration of a Model of the Blood-Brain Barrier (BBB)

**Figure 8:**
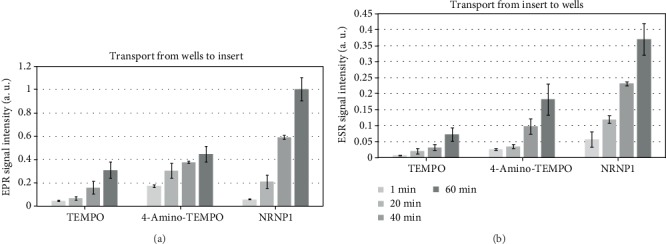
Two-directional transport of nitroxides and NRNP1 (from the basolateral to the apical compartment (a) and from the apical to the basolateral compartment (b)) across the model of the blood-brain barrier.: *n* = 9.

NRNP1, like TEMPO and 4-amino-TEMPO, penetrated the model BBB of hCMEC/D3 cells. The transport took place in both directions at comparable rates, indicating the lack of an active transporter (Figure 8).

#### 3.2.4. ATP Level

**Figure 9:**
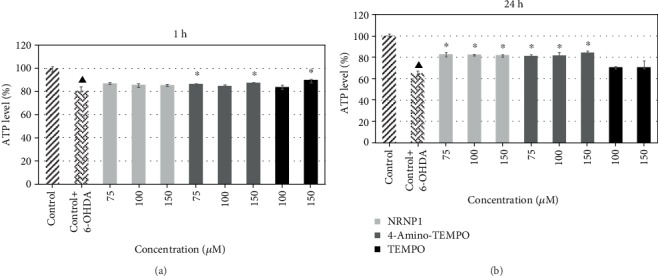
ATP levels after treatment with selected antioxidants and 6-hydroxydopamine after 1 (a) and 24 hours (b). The whiskers are in lower (25%) and upper (75%) quartile ranges. ^∗^*P* ≤ 0.05, Kruskal–Wallis test against the 6-OHDA-treated control. ^▲^*P* ≤ 0.05 differences between controls; *n* = 9.

A decrease in intracellular ATP level was observed even after 1 h incubation with 6-OHDA, but about 10% improvement was seen in samples preincubated with antioxidants. Nitroxides and NRNP1 alone did not induce significant changes. However, a long-term exposure to 6-OHDA reduced ATP level to about 65%, whereas both NRNP1 and 4-amino-TEMPO exhibited a similar effect, i.e., an about 20% protection (Figure 9).

#### 3.2.5. Glutathione Level

**Figure 10:**
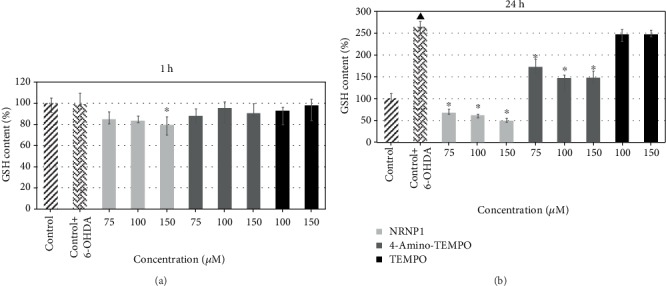
GSH content after treatment with selected antioxidants and 6-hydroxydopamine after 1 (a) and 24 hours (b). The whiskers are in lower (25%) and upper (75%) quartile ranges. ^∗^*P* ≤ 0.05, Kruskal–Wallis test against the 6-OHDA-treated control. ^▲^*P* ≤ 0.05 differences between controls; *n* = 9.

The effects of 6-OHDA on GSH content in SH-SY5Y cells without and after 2 h treatment with NRNP1 and free nitroxides were examined after 1 and 24 h incubation. Short-term (1 h) incubations with 6-OHDA did not statistically significantly affect GSH content. In contrast, 24 h incubation with 6-OHDA induced a significant increase in the GSH content (Figure 10).

Preincubation with nitroxides or NRNPs followed by 6-OHDA treatment did not affect significantly the GSH level except for a small decrease at 150 *μ*M NRNPs after short-term incubation. After 24 h treatment, amelioration of the increase in the GSH level was seen for 4-amino-TEMPO, and a complete abolition of the increase with even some decrease below the control level was noted for NRNPs. TEMPO showed no significant effect on the 6-OHDA-induced changes in the GSH level (Figure 10).

#### 3.2.6. Level of Reactive Oxygen Species

**Figure 11:**
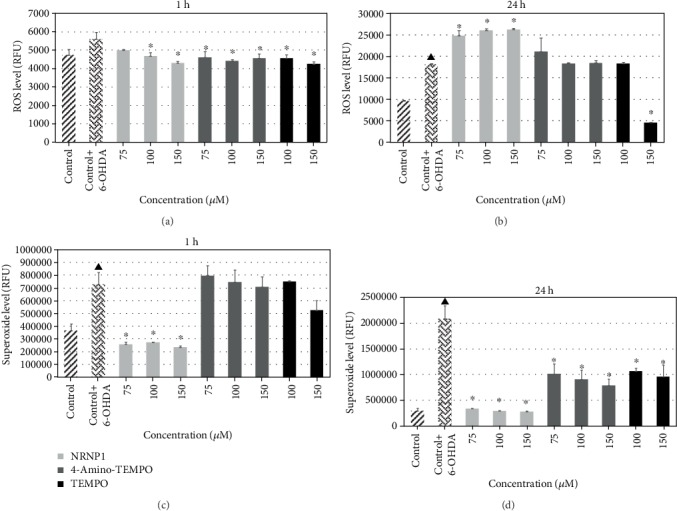
ROS level estimated with DHE after treatment with selected antioxidants and 6-hydroxydopamine for 1 (a) and 24 hours (b) and superoxide level after treatment with selected antioxidants and 6-hydroxydopamine for 1 (c) and 24 hours (d). The whiskers are in standard deviation. ^∗^P ≤ 0.05, Student *t*-test against the 6-OHDA-treated control. ^▲^*P* ≤ 0.05 differences between the controls; *n* = 3.

A slight rise in ROS level measured with DHE, which shows some specificity towards O_2_^·-^, was observed after 1 h incubation with 6-OHDA, and a significant increase after 24 h was also observed. All of the studied antioxidants decreased the ROS level, except NRNP1 at 75 *μ*M concentration (Figure 11(a)). While after 24 h, the nanoparticles further increased ROS level, the level of ROS was unchanged in the presence of free radicals except for the highest concentration of TEMPO, which considerably decreases the level of ROS (Figure 11(b)). 6-OHDA exposure caused a substantial increase in the mitochondrial superoxide level, when measured with O_2_^·-^-specific probe, MitoROS™. Free nitroxides counteracted this effect very efficiently even at the lowest concentration (75 *μ*M) (Figures 11(c) and 11(d)), whereas NRNP1 attenuated this effect only after 24 h (Figure 11(d)).

#### 3.2.7. Mitochondrial Membrane Potential

**Figure 12:**
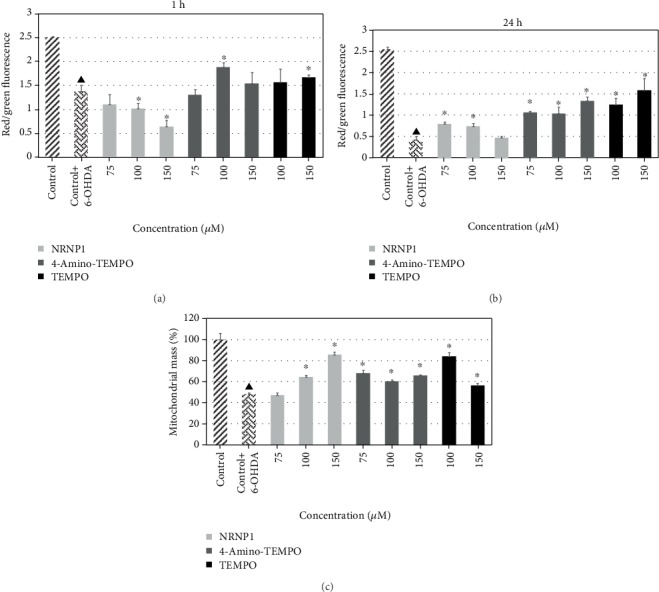
Changes in the mitochondrial potential after treatment with selected antioxidants and 6-hydroxydopamine for 1 (a) and 24 hours (b) and changes in the mitochondrial mass after treatment with selected antioxidants and 6-hydroxydopamine for 24 hours (c). The whiskers are in standard deviation. ^∗^*P* ≤ 0.05 Student *t*-test against the 6-OHDA-treated control. ^▲^*P* ≤ 0.05 differences between the controls; *n* = 3 (a, b) and 9 (c).

We estimated mitochondrial membrane potential (Δ*ψ*_m_) changes by JC-1 staining of SH-SY5Y cells treated with various concentrations of NRNP1 and free nitroxides. The results demonstrated that mitochondrial membrane potential was significantly reduced in 6-OHDA-treated cells (after short-term and long-term treatment). After short-term exposure to 6-OHDA, NRNP1 did not exhibit a protective effect in this manner; there was even a further decrease in Ψ_m_ at 100 and 150 *μ*M concentrations of NRNPs. Only 4-amino-TEMPO and TEMPO at 100 *μ*M and 150 *μ*M concentrations, respectively, showed some preventive effect. After long-term treatment, a considerable reduction in Ψ_m_ was observed, where selected antioxidants at nearly all concentrations slightly exhibited a protective effect, except NRNPs at 150 *μ*M concentration (Figures 12(a) and 12(b)).

#### 3.2.8. Mitochondrial Mass

The long-term incubation with 6-OHDA caused a significant decrease in the mitochondrial mass up to 50% compared to the nontreated control. NRNP1 (except 75 *μ*M concentration), 4-amino-TEMPO, and TEMPO hampered this effect; yet, 150 *μ*M concentration of NRNP1 and 100 *μ*M of TEMPO evoked the most effective outcome. Other antioxidants attenuated changes in the mitochondrial mass at a similar level, producing an about 20% increase compared to the 6-OHDA-treated control (Figure 12(c)).

#### 3.2.9. MitoTracker Labelling

Cultures of SH-SY5Y cells treated as described in Section 2.7.2 were stained with MitoTracker Deep Red FM and phalloidin, which target the intracellular mitochondrial network and cytoskeletal actin filaments, respectively. In addition, cell nuclei were simultaneously stained with DAPI (targeting DNA in the cell nucleus; ultraviolet excitation with blue emission) (Figure 2(a)). The ratio of fluorescence intensity of mitochondria and cytoskeleton (expressed as mean gray value) was calculated using ImageJ software. The analysis shows an about 20% decrease in the signal ratio between the 6-OHDA-treated and untreated controls. There is an about 20% higher signal ratio in a sample treated with 150 *μ*M NRNP1 and an about 20% lower when treated with 150 *μ*M 4-amino-TEMPO compared to the 6-OHDA-treated control sample (Figure 2(b)). TEMPO, however, gives a similar signal ratio with the 6-OHDA-treated control. These results well correlated with the results of mitochondrial mass estimation. The cells treated with 6-OHDA have evenly scattered mitochondria compared to the control cells. In cells treated with NRNP1, this tendency is also observed, but there are some clusters of mitochondria visible. In other samples, mitochondria are more concentrated in proximity to cellular membrane, which correlates with untreated control.

## 4. Discussion

It should be emphasized that current PD treatments ameliorate only the symptoms of the disease without halting its progress. Finding new medicines inhibiting the development of this disease can open new perspectives for the patients and considerably reduce healthcare expenses. Oxidative stress inevitably accompanies PD. The employment of antioxidants in this disease has been proposed to ameliorate OS and its consequences [50–54]. Nevertheless, the effects of antioxidants, among them natural components of the diet are limited and new, more efficient antioxidants are searched for. Redox nanoparticles, polymers containing covalently linked nitroxyl radicals, showing low toxicity, and good stability *in vivo*, seem to be promising candidates for ameliorating OS in PD [55].

6-Hydroxydopamine treatment of neuronal cells is an acknowledged cellular model of PD. The damage of cells exposed to 6-OHDA has been ascribed to several mechanisms, including generation of ROS by autoxidation of 6-OHDA and mitochondrial damage, which may also lead to increased ROS production in the mitochondria [53, 56, 57]. We did not observe a significant generation of ROS by 6-OHDA in the absence of cells under tissue culture conditions. 6-OHDA produced low amounts of H_2_O_2_ in the medium, but at the same time, it showed also antioxidant properties, scavenging ABTS^∗^ in a fast and in a slow reactions, its fast reactivity being similar to tyrosine and tryptophan, albeit lower (Table 2). However, 6-OHDA increased the intracellular levels of cytoplasmic and mitochondrial ROS (mainly superoxide) as estimated with DHE and MitoROS™ 520 (Figures 11(a) and 11(b)). 6-Hydroxydopamine exhibited a dose-dependent toxicity against SH-SY5Y cells (Figure 5). Nitroxide radical-containing redox nanoparticles as well as free nitroxides, TEMPO, and 4-amino-TEMPO protected against 6-OHDA toxicity, NRNP1 being the most effective on the (segment) molar basis (Figure 6(b)). The compounds studied, when administered alone, were slightly cytotoxic at the highest concentrations applied (Figure 6(a)); though, when applied together with 6-OHDA, they were protective. Apparently, the protective effect of NRNPs and nitroxides is due to their antioxidant properties. Antioxidant properties of NRNPs have been described in the literature [41, 43]. We demonstrated protective effects of NRNPs against oxidation by SIN-1 chloride and protection of fluorescein against bleaching by AAPH and hypochlorite (Table 1).

6-Hydroxydopamine increased the level of cytoplasmic ROS (mainly superoxide) after 1 h and 24 h incubation. An increase in the intracellular ROS level in the 6-OHDA-treated cells has been also reported by other authors [55]. While the increase after 1 h was attenuated by NRNP1 and free nitroxides (Figure 11(a)), the ROS increase after 24 h was not affected by free nitroxides except for the highest concentration of TEMPO, but augmented by NRNP1 (Figure 11(b)). The 6-OHDA-induced increase in the superoxide level was most pronounced in the mitochondria. In this case, NRNP1 showed a strong protective effect, while free nitroxides was effective only after 24 h (Figures 11(c) and 11(d)). The apparent increase in the ROS level in the presence of NRNP1 (Figure 11(b)) is surprising; we attribute it to the direct oxidation of the fluorogenic probe by NRNP1 observed by us [58].

Of critical importance for any substance considered for having a possible effect on the course of PD is its ability to penetrate the BBB. Brain endothelial cells (BECs) forming the BBB express the adenosine triphosphate-binding cassette efflux transporters, P-glycoprotein (P-gp), and the breast cancer resistance protein (BCRP), which have a similar substrate overlap, and act to prevent toxins and unwanted blood-borne signaling molecules from entering the brain [59]. To date, there have been no reported investigations regarding the assessment of BBB penetration by NRNPs and free nitroxides in an *in vitro* model employing human brain capillary endothelium cells hCMEC/D3 [44, 47]. Our results show that NRNP1 are able to penetrate the BBB, judging from the penetration of the BBB model formed by hCMEC/D3 cells. Two-directional transport at comparable rates indicates penetration of the barrier not mediated by an active transporter. Basing on the EPR signal intensity, the transport of NRNP1 was faster than that of free nitroxides as per amount of free radical residues (Figure 8), indicating a possible transcellular vesicular transport component.

The level of glutathione was not significantly altered by 6-OHDA after 1 h incubation and significantly increased after 24 h. Apparently, this is a compensative effect being a response to a rapid GSH depletion by 6-OHDA (not visualized by our studies) due to the activation of GSH biosynthesis. Such an effect has been described previously for SH-SY5Y cells [60]. These authors concluded that 6-OHDA induced a concentration-dependent increase in GSH and total glutathione concentrations after 24 h. After exposure to 50 *μ*M 6-OHDA, GSH concentrations were increased up to 12-fold after 24 h with no change in the GSH : GSSG ratio. Gene analysis suggested that the increase in GSH concentration was due to the increased expression of the GSH biosynthesis genes, coding for glutamate cysteine ligase modifier and catalytic subunits. To resume, 6-OHDA induces OS in SH-SY5Y cells resulting in an adaptive increase in cellular GSH concentrations. NRNP1 and 4-amino-TEMPO prevented this effect (Figure 10).

It should be noted that nitroxides also react with GSH [58] and thus may induce a compensative GSH biosynthesis in cells exposed to nitroxides as well as after 24 h; in the case of NRNP1, this effect was not observed, most probably due to different reactivity of NRNP1 with GSH, with respect to free nitroxides (Figure 10). Previous studies pointed to a much lower reactivity of NRPN1 with respect to free nitroxides for another reducing agent, ascorbate [41].

6-Hydroxydopamine induced a decrease in the cellular ATP content, apparently due to mitochondrial dysfunction. This decrease was counteracted by NRNP1 and free nitroxides (Figure 9). Indeed, 6-OHDA induced mitochondrial depolarization as detected by the JC-1 probe, which was attenuated by NRNP1 and nitroxides after 24 h incubation. A decrease in the mitochondrial mass was also observed in the 6-OHDA-treated cells; this decrease was prevented by NRNP1 and nitroxides (Figure 12). Similar direction of changes was seen when the intensity of mitochondrial staining with MitoTracker Red was compared (Figure 2). This phenomenon might be correlated with the fact that several studies have shown that 6-OHDA affects mitochondrial fusion and fission causing an imbalance in mitochondrial dynamics, and it is proven that it may contribute to the pathogenesis of neurodegenerative diseases [61–63]. Previous studies demonstrated that 6-OHDA (albeit at a high concentration of 150 *μ*M) caused a loss of cell viability and mitochondrial depolarization [55, 64].

It can be suggested on the basis of the presented results that the main effect of 6-OHDA on SH-SY5Y cells consists of the mitochondrial damage. 6-Hydroxydopamine, as a positively charged compound, can be expected to accumulate in the mitochondria and increase ROS production primarily in the mitochondria. The resulting mitochondrial damage can lead to the depolarization of the inner mitochondrial membrane, a decrease in the mitochondrial mass, and diminution of ATP production, resulting in many site cellular damage. Mitochondrial damage by 6-OHDA was attributed to two mechanisms: ROS production and inhibition of the mitochondrial respiratory chain complexes I and IV [65]. As both ROS and reactive nitrogen species (RNS) were implicated in the 6-OHDA damage to nigrostriatal dopaminergic neurons seen in PD [66], it should be emphasized that nitroxyl radicals (and NRNPs) react with both ROS and RNS, being able of catalytic decomposition of peroxynitrite [32]. Another factor involved in the 6-OHDA cytotoxicity is iron, especially Fe^2+^ contained in the intracellular labile iron pool; iron chelators reducing this pool protect SH-SY5Y cells against 6-OHDA toxicity [67]. Nitroxides and NRPNs are able to oxidize Fe^2+^ ions, eliminating their activity in free radical reactions, and this effect may also contribute to their protective action. Thus, nitroxide radical-containing redox nanoparticles are effective compounds for the protection of neuronal cells against the 6-OHDA-induced damage, and as they are more stable *in vivo* and less prone to reduction than free nitroxides [40, 54], they could be promising as agents inhibiting the neuronal damage in PD.

## 5. Conclusion

Our results suggest that the mitochondria are the main site of 6-OHDA-induced cellular damage and demonstrate a protective effect of NRNP1 in a cellular model of PD. Understanding the protective effects of NRNP1 against PD progress requires further careful *in vivo* investigation.

## Figures and Tables

**Table 1:** Protection against dihydrorhodamine 123 oxidation by SIN-1: and against fluorescein bleaching by hypochlorite and AAPH (IC_50_ values).

Compound.	Protection against DHR 123 oxidation (IC_50_, *μ*M)	Protection against fluorescein bleaching by NaOCl (IC_50_, *μ*M)	Protection against fluorescein bleaching by AAPH (IC_50_, *μ*M)
NRNP1	0.98 ± 0.06	2.11 ± 0.12	0.56 ± 0.03
NRNP2	0.25 ± 0.02	1.52 ± 0.06	1.45 ± 0.08
4-Amino-TEMPO	3.35 ± 0.13	5.48 ± 0.14	7.85 ± 0.15

**Table 2:** Reducing activity of the 6-hydroxydopamine, antioxidant amino acids, and standard antioxidants (ABTS^∗^ assay) and generation of hydrogen peroxide in DMEM/F12 medium.

Compound.	Fast ABTS^∗^ scavenging activity (Mol TE/Mol)	Slow ABTS^∗^ scavenging activity (Mol TE/Mol)	H_2_O_2_ (*μ*M)
6-Hydroxydopamine	0.172 ± 0.006	0.201 ± 0.006	7.8 ± 1.5
Tyrosine	1.845 ± 0.063	—	—
Tryptophan	1.883 ± 0.074	—	—
Ascorbic acid	1.114 ± 0.017	—	33.6 ± 0.9
Glutathione	1.027 ± 0.004	—	nd^∗^

mean ± SD; *n* ≥ 3 (independent samples). and^∗^: not detectable amounts; TE: Trolox equivalents.

## Data Availability

Data available on request. Please contact I. Sadowska-Bartosz, e-mail: isadowska@poczta.fm.

## Abbreviations

4-amino-TEMPO: 4-Amino-2,2,6,6-tetramethylpiperidine-1-oxyl
6-OHDA: 6-Hydroxydopamine
AAPH: 2,2-Azobis (2-amidinopropane) dihydrochloride
ABTS^∗^: 2,2′-Azinobis (3-ethylbenzthiazoline-6-sulfonic acid) radical
BBB: Blood-brain barrier
DHE: Dihydroethidium
DHR123: Dihydrorhodamine 123
DMEM/F12: Dulbecco's Modified Eagle Medium Nutrient Mixture F-12
DMSO: Dimethyl sulfoxide
Drp1: Dynamin-related protein-1
DTPA: Diethylenetriaminepentaacetic acid
EBM-2: Endothelial cell growth basal medium-2
ESR: Electron spin resonance
FBS: Foetal bovine serum
GSH: L-Glutathione reduced
JC-1: 5,5′,6,6′-Tetrachloro-1,1′,3,3′-tetraethylbenzimidazolylcarbocyanine iodide
Mohr's salt: Fe (NH_4_)_2_(SO_4_)_2_
MTT: 3-(4,5-Dimethylthiazol-2-yl)-2,5-diphenyltetrazolium bromide
NAO: Acridine orange 10-nonyl bromide
NEM: *N*-Ethylmaleimide
NRNPs: Nitroxide-containing redox nanoparticles
OPA: *ortho*-Phthalaldehyde
OS: Oxidative stress
Parkin: Parkinson juvenile disease protein 2
PBS: Phosphate-buffered saline
PD: Parkinson's disease
PEG-b-PCTEMPO: A self-assembling amphiphilic block copolymer composed of a hydrophilic poly (ethylene glycol) (PEG) segment and a hydrophobic poly (chloromethylstyrene) (PCMS)
PINK1: PTEN-induced kinase-1
RNS: Reactive nitrogen species
ROS: Reactive oxygen species
SEM: Scanning electron microscopy
SIN-1: 5-Amino-3-(4-morpholinyl)-1,2,3-oxadiazolium chloride
TCA: Trichloroacetic acid
TE: Trolox equivalents
TEMPO: 2,2,6,6-Tetramethylpiperidine-1-oxyl
TEMPOL: 4-Hydroxy-2,2,6,6-tetramethylpiperidine-1-oxyl
Δ*ψ*_m_: Mitochondrial membrane potential
IC_50_: Half maximal inhibitory concentration.
